# Nitrogen-Doped Carbon Dots as a Fluorescent “Off–On” Probe for Selective Ascorbic Acid Detection via H_2_O_2_-Mediated Quenching

**DOI:** 10.3390/nano15130976

**Published:** 2025-06-23

**Authors:** Jingjing Jia, Xue Liu, Wenjing Wang

**Affiliations:** College of Chemistry and Chemical Engineering, Shandong Sino-Japanese Center for Collaborative Research of Carbon Nanomaterials, Instrumental Analysis Center, Qingdao University, Qingdao 266071, China; 2022020328@qdu.edu.cn (J.J.); 2023020334@qdu.edu.cn (X.L.)

**Keywords:** carbon dots, H_2_O_2_, AA detection, luminescence mechanism, fluorescent “off–on” probe

## Abstract

Nitrogen-doped carbon dots (NCDs) exhibiting superior fluorescence characteristics were synthesized employing o-phenylenediamine and 2-methylimidazole as precursors. The synthesized NCDs exhibited yellow photoluminescence with an excitation/emission maxima of 410/554 nm with a quantum yield of 28.41%. The presence of pyridinic N, pyrrolic N, graphitic N, and amino N functionalities on the NCDs’ surface provided strong evidence for the successful nitrogen doping of the carbon dots. Upon exposure to hydrogen peroxide (H_2_O_2_), the NCDs exhibited a significant reduction in fluorescence intensity, which could be restored by the addition of ascorbic acid (AA), demonstrating a quantitative relationship between ascorbic acid and fluorescence efficiency. A novel fluorescence “off–on” system utilizing these NCDs was developed for the quantification of AA. The sensing mechanism relies on H_2_O_2_-induced fluorescence quenching via the selective oxidation of the NCDs’ surface, followed by fluorescence restoration upon AA addition due to the reduction in surface defects. Meanwhile, further experiments confirmed that the quenching mechanism was static quenching. The NCDs demonstrated a limit of detection (LOD) of 0.605 μM for AA detection. The use of NCDs for AA sensing was validated through the analysis of commercially available beverages. This study aimed to establish a simplified method for ascorbic acid detection. The experimental findings indicated that the developed technique exhibited high accuracy in quantifying ascorbic acid. These findings suggest that the developed NCDs possess considerable potential as a multifunctional sensing tool for various analytical applications.

## 1. Introduction

Carbon dots (CDs) are a nanomaterial with quasi-spherical morphology; generally, their size is between 1 and 10 nm. Their structure constitutes aromatic carbon nuclei and a hybridized sp^2^/sp^3^ carbon lattice [1]. Carbon dots have various characteristics, such as tunable optical properties, good biocompatibility, high photostability, and low-cost synthesis. Therefore, the application of CDs is extensive, such as in ionic detection, solar energy conversion, nano-thermometry, biomedical applications, and biological imaging [2,3,4,5]. However, diverse precursors and synthetic methods are needed for CDs, thereby limiting their broader applicability. However, pristine CDs often suffer from limitations such as relatively low quantum yields and sensitivity to environmental conditions, thereby hindering their broader applicability.

Heteroatom doping has emerged as a particularly effective method to overcome these limitations and enhance the optical performance of CDs [6]. Doping carbon dots with heteroatoms (e.g., nitrogen, sulfur, boron, etc.) is a highly effective strategy for enhancing and modifying their inherent optical properties [7]. These dopant atoms enable the precise modulation of carbon dots, leading to tailored fluorescence characteristics. Among them, nitrogen (N) atoms are the most commonly used dopants, due to the comparable atomic sizes of carbon and nitrogen, which facilitates their integration within the carbon structure. The presence of five valence electrons in nitrogen facilitates the electrons’ injection into the carbon structure [8]. Sulea Kellici’s team used a theoretical computational model to investigate the impact of different N-containing precursors on the characteristics of NCDs synthesized through continuous hydrothermal flow synthesis [9]. Similarly, Zhong’s team employed phenylenediamine and phthalic anhydride as precursors to synthesize NCDs through a simple method, focusing on elucidating the relationship between fluorescent functional groups and the carbon core structure in controlling or adjusting the molecular configuration of the CDs [10]. Su’s team used lemon peel as a precursor for NCD synthesis and employed Cu^2+^ in aqueous solutions through dynamic quenching. The NCDs showed a good fluorescence response and strong resistance to photobleaching. Under optimal experimental conditions, a good linear relationship was established. This method is reliable and efficient and provides a new idea for clinical analysis [11]. Aparicio’s team synthesized pyrolytic lignin-based carbon dots (CDPLs) via controlled thermal pyrolysis in water. The one-pot method produced blue/green fluorescent CDPLs with an average size of 34 nm. The characterization techniques revealed the optical properties and chemical composition of CDPLs, with a fluorescence quantum yield of 7.9%, which is comparable to those of lignin-derived carbon dots [12]. However, despite the numerous synthesis methods available and the variety of nitrogen-containing precursors employed, the complex relationship between the precursor’s chemical structure and electronic structure of nitrogen-doped carbon dots needs further investigation [7,13,14].

The remarkable fluorescence characteristics of NCDs have fueled their widespread exploration as fluorescent sensors, establishing them as a prominent research area. The sensing mechanism of NCD-based fluorescent sensors relies on the modulation of their fluorescence emission (“off–on” or “on–off”) upon interaction with the target analyte, resulting in a concentration-dependent response [15]. Direct fluorescence quenching, however, can introduce inaccuracies and compromise analytical performance. To mitigate these limitations, researchers have explored the combination of NCDs with external fluorescence quenchers (such as Fe^3+^, Hg^2+^, Cu^2+^, and MnO_4_^−^) in the development of the “switch on” sensors [16]. These sensors, which exhibit analytical sensitivity, have been applied in the detection of environmental pollutants, biomolecules, food components, etc.

Ascorbic acid (AA) is an essential micronutrient recognized for its powerful antioxidant properties. It has a vital role in various biological functions, including cellular growth, metabolic regulation, and modulation of the immune response [17]. Since humans cannot synthesize ascorbic acid internally, sufficient dietary intake is required to ensure optimal health and well-being. AA acts as a reducing agent in biological redox reactions, protecting against oxidative stress and supporting enzyme function [18]. A lack of AA can cause scurvy and increase susceptibility to infections, and it could play a role in the progression of chronic conditions [16]. Fruits and vegetables are the main dietary sources of AA, with many commercial beverages also being fortified with vitamin C. This creates a demand for accurate and convenient quantification methods. Numerous analytical methods have been used to detect AA, including electrochemical approaches, chromatography, titrimetry, and spectrophotometry [19]. However, most of these technologies suffer from limitations such as cumbersome operations, high cost, and complex instrumentation. Fluorescent analysis technology offers a compelling alternative owing to its exceptional sensitivity, real-time detection potential, excellent reproducibility, and user-friendly operation.

The accurate measurement of both antioxidant and oxidant species is essential across a wide spectrum of fields, from ensuring food quality to improving clinical diagnoses. While AA functions as a significant antioxidant, the detection of oxidants, like hydrogen peroxide (H_2_O_2_), is of equal importance. H_2_O_2_ is a relatively common peroxide compound widely employed as an oxidant and a bleaching and disinfecting agent in various industrial and consumer applications [20]. While H_2_O_2_ readily decomposes into water and oxygen under typical conditions, its presence and concentration in various environments are of considerable interest. Under normal circumstances, H_2_O_2_ readily undergoes decomposition into water and oxygen, making it unstable and difficult to persist over extended periods. While mildly elevated levels of H_2_O_2_ can promote tumor development by fostering cancerous cell transformation, proliferation, and survival, as well as angiogenesis and metastasis, its high concentrations induce cytotoxic effects through damage to essential biomolecules [21]. Consequently, the creation of fast and precise analytical techniques for H_2_O_2_ detection continues to be a critical focus in the fields of food safety and clinical diagnostics.

In this work, we synthesize NCDs exhibiting strong yellow photoluminescence (λex/λem = 410/554 nm, quantum yield of 28.41%) and surface functionalities indicative of successful nitrogen doping, aiming to further elucidate the relationship between N-containing precursor structures and the resulting optical properties of NCDs. These NCDs were then utilized as a fluorescence sensor for AA detection. The sensing process relies on the reversible quenching of NCD fluorescence by H_2_O_2_, followed by fluorescence recovery upon AA addition, facilitating the quantitative detection of AA. Furthermore, the applicability of this NCD-based sensor was validated through AA quantification in commercially available beverages. These results highlight the potential of these NCDs as a promising and versatile platform for fluorescence-based analytical applications.

## 2. Materials and Methods

### 2.1. Materials and Apparatus

*O*-phenylenediamine (OPD) and 2-methylimidazole were procured from Aladdin Ltd. (Shanghai, China). H_3_PO_4_, NaOH, H_2_O_2_, H_3_BO_3_, glutathione, ascorbic acid, chitosan, sodium phosphate, ferric nitrate, chromium nitrate, manganese nitrate, copper nitrate, nickel chloride hexahydrate, 2,3-diaminophenazine, and deuterated dimethyl sulfoxide (DMSO) were obtained from Sinopharm Chemical Reagent Co. Ltd., Shanghai, China. Furthermore, the water utilized was secondary deionized water, which required no additional purification steps.

The examination was conducted using high-resolution transmission electron microscopy (HRTEM) on an FEI Tecnai G2 F20 system (Philips, Eindhoven, Netherlands). Using a Nicolet iS50 spectrometer (Thermo Fisher Scientific, Waltham, MA, USA), Fourier-transform infrared (FTIR) spectra were acquired for molecular vibrational investigations. Its optical characteristics were evaluated by utilizing an F-7000 fluorescence spectrophotometer (Hitachi, Tokyo, Japan) to measure fluorescence emission spectra. A DXR2 Raman spectrometer (Thermo Fisher Scientific, Waltham, MA, USA) was utilized for molecular structure analysis, a UV-2700 spectrophotometer (Shimadzu, Tokyo, Japan) was used for ultraviolet–visible (UV–vis) spectroscopy to analyze optical absorption characteristics and to determine the chemical composition of the surface, and X-ray photoelectron spectroscopy (XPS) measurements were performed using an ESCALAB 250XI spectrometer (Thermo Fisher Scientific, Waltham, MA, USA). A Smart Lab 3 KW X-ray diffractometer (Rigaku Corporation, Tokyo, Japan) was used to obtain the X-ray diffraction (XRD) analysis. Avance III 400 MHz Nuclear Magnetic Resonance (Bruker, Biel, Switzerland) was used to obtain the ^1^H NMR and ^13^C NMR spectra. An FLS 980 steady/transient fluorescence spectrophotometer (Edinburgh Instruments, Edinburgh, UK) was used to measure the fluorescence lifetime spectra.

### 2.2. Synthesis of NCDs

**Scheme 1:**
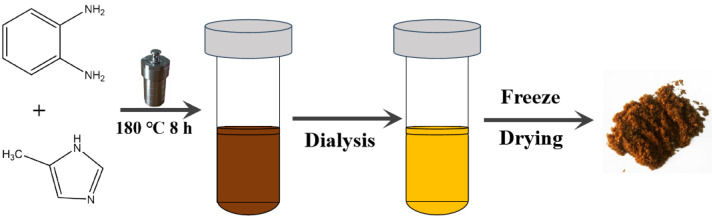
The one-pot hydrothermal synthesis and purification of NCDs.

A mixture containing 0.5 g of o-phenylenediamine and 0.5 g of 2-methylimidazole was dissolved in water. This synthesis was carried out at 180 °C for 8 h. After the completion of the reaction, the autoclave was left to cool naturally to room temperature. Carbon dots (CDs) were then subjected to purification through dialysis by using a cellulose ester dialysis membrane (Shanghai Yuanye Bio-Technology Co., Ltd., Shanghai, China, MWCO: 100–500) with ultrapure water for 24 h to remove residual reactants and low-molecular-weight impurities. The purified nitrogen-doped carbon dots (NCDs) were collected at room temperature for subsequent applications. For long-term storage and characterization purposes, an aliquot of the NCD solution was freeze-dried to produce a powder, which was subsequently pulverized into fine particles. The resulting carbon dot powder was preserved at 4 °C for potential future utilization. The synthesis and processing procedure of NCDs are illustrated in Scheme 1.

### 2.3. QY of NCDs

The specific experimental procedure of NCDs is as follows: Rhodamine B was chosen as the reference fluorophore, and it was dissolved in an ethanol solution under ambient conditions, exhibiting a fluorescence quantum yield of 56%. The ethanolic solutions of NCDs and RhB were prepared at concentrations adjusted to ensure UV absorbance values under 0.05 at 410 nm, thereby reducing inner filter effects. Subsequently, the fluorescence emission spectra of NCDs and the RhB solution were measured. Each measurement was performed in triplicate. The integrated fluorescence emission spectral area for each replicate was calculated and plotted against the corresponding absorbance value. Linear regression was performed on the resulting data, and the slope (K) of the fitted line was determined. Substituting the slope K into the following equation yields the quantum yield: ${\Phi_{x}=\Phi_{st}\left(K_{x}/K_{st}\right)\left(\eta_{x}/\eta_{st}\right)}^{2}$. Φ denotes the QY; η represents refractive indices (1.36 for ethanol and 1.33 for water); K represents the slope; lower marks “x” and “st” correspond to NCDs and RhB, respectively.

### 2.4. Detection of H_2_O_2_

A 200 μL volume of carbon dot solution (5 μg·mL^−1^), 500 μL of hydrogen peroxide solution of different concentrations (0.5–3 μM), and 1.30 mL of water were added to several centrifuge tubes. The fluorescence intensity of the mixture at λex/λem (410 nm/554 nm) was recorded following a 20 min incubation period at ambient temperature, denoted as F. A control group was prepared, hydrogen peroxide was replaced with an equal volume of ultrapure water, and fluorescence intensity was denoted as F_0_. A calibration curve was constructed by correlating the concentration of the H_2_O_2_ solution and the fluorescence intensity of NCDs.

### 2.5. The Procedure of AA Detection

Various concentrations of AA (1–30 μM) reacted with the NCDs/H_2_O_2_ system. After 20 min of incubation at room temperature, fluorescence intensities under excitation at 410 nm were recorded, denoted as F. A control (F_0_) was prepared with ultrapure water in place of AA.

To investigate the selectivity of the NCDs, potential interference from various common substances was assessed. The following compounds were selected as potential interferents: glutathione, ascorbic acid, chitosan, H_2_O_2_, PO_4_^3−^, BO_3_^3−^, Fe^3+^, Cr^3+^, Cu^2+^, Ni^+^, and Mn^2+^. Each potential interfering substance was evaluated at a concentration of 500 μM and was recorded using 410 nm excitation.

### 2.6. Analysis of Real Sample

To investigate the NCD-based assay for application in real-world sample analysis, the AA content of several commercially available beverages was determined. The following beverages were purchased from local retailers: lemon soda, Alien Vitamin C water, Minute Maid orange juice, and water-soluble C100. Prior to analysis, the beverage samples were processed using filtration and centrifugation to remove impurities. For each measurement, 1 mL of H_2_O_2_ solution (3 μM) and 500 μL of NCD solution (5 μg mL^−1^) were transferred in sequence to a 5 mL centrifuge tube. After achieving uniform mixing, a defined concentration of AA standard solution along with 500 μL of the beverage sample were transferred to the tube. Following a 20 min incubation at room temperature, the fluorescence emission of NCDs/H_2_O_2_/AA at λex/λem (410 nm/554 nm) was measured. All samples were analyzed in triplicate.

## 3. Results and Discussion

### 3.1. Preparation Optimization

The optimization of NCDs’ hydrothermal synthesis was conducted by studying the impact of reaction time and temperature. First, the reaction time was varied while maintaining a constant temperature of 180 °C. The fluorescence emission spectra of NCD solutions were measured at 410 nm excitation for samples synthesized under different reaction durations. In Figure S1a, the maximum fluorescence emission was observed for NCDs synthesized in 8 h, and the lowest fluorescence intensity was obtained after 4 h of reaction. Therefore, an 8 h reaction period yielded the best carbonization results.

Next, we examined how tweaking the synthesis temperature, specifically between 140 °C and 220 °C, impacted fluorescence intensity, all while keeping the reaction time pegged at a steady 8 h. In Figure S1b, the optimal fluorescence emission was observed at a carbonization temperature of 180 °C. The synthesis of NCDs was found to be most effective under the conditions of a reaction temperature of 180 °C and a reaction time of 8 h. Composites have been successfully prepared in this manner.

### 3.2. NCD Characterization

**Figure 1:**
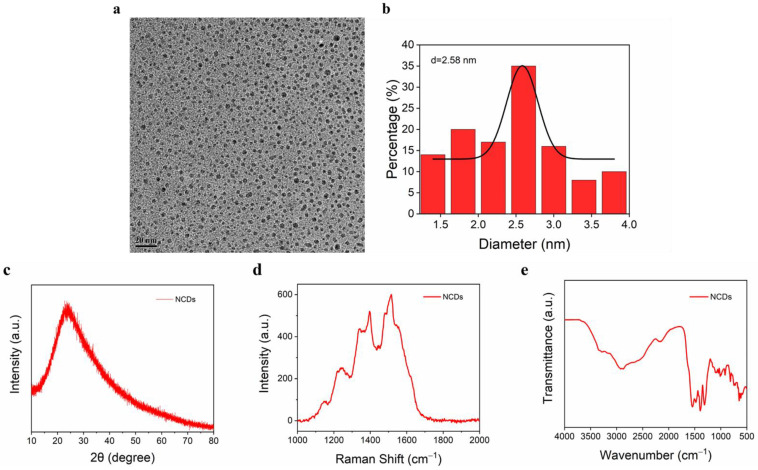
The HRTEM images of the synthesized NCDs (scale bar = 20 nm) (**a**), the particle size distribution histogram of the NCDs derived from the TEM images, indicating an average particle size of 2.58 nm (**b**), the Raman spectrum of NCDs (**c**), the XRD spectra of NCDs (**d**), and the FT-IR spectra of the prepared NCDs (**e**).

High-resolution transmission electron microscopy served as a primary tool for evaluating NCDs’ morphology. The prepared NCDs are evenly distributed in an aqueous solution without obvious aggregation. NCDs present a uniform quasi-spherical shape, and the particle size is less than 5 nm, with an average particle size determined to be 2.58 nm (Figure 1a,b). Compared with the hydrothermal synthesis of carbon dots in the literature review of M.C.M.D. de Conti’s team [22], they have reported the same structural morphology and size of the carbon dots. The obtained CDs demonstrate a uniform size, a good spherical shape, and high dispersibility, matching the structural criteria reported for the excellent performance of carbon dots. The chemical structure and surface composition of NCDs were further studied using various characterization methods. The XRD of NCDs exhibits a broad peak centered at 24.3° (Figure 1d), which is defined as the characteristic diffraction peak (002) of the graphite structure, indicating the presence of a graphitic carbon structure within the NCDs. The Raman spectrum of the NCDs displays two prominent peaks at 1394 cm^−1^ (D band) and 1530 cm^−1^ (G band) (Figure 1c), which are representative of sp^2^-hybridized and sp^2^-hybridized graphitic carbon structures. A common indication of the degree of graphitization is the I_D_/I_G_ ratio, which is the ratio of the D band intensity to the G band intensity. The calculated I_D_/I_G_ value for the synthesized NCDs was 0.73. The degree of graphitization increases as the I_D_/I_G_ ratio decreases.

The FT-IR spectra show several different absorption bands, which represent the surface element composition of NCDs. The stretching vibrations of C=C/C=O, C-O, and C-N are represented by peaks located approximately at 1607 cm^−1^, 1316 cm^−1^, and 1121 cm^−1^ (Figure 1e), respectively. The pronounced intensity of the peaks at 1607 cm^−1^ and 1121 cm^−1^ indicates significant oxidation and nitrogen incorporation. A broad absorption band spanning 3717–3144 cm^−1^ is assigned to the stretching vibrations of O-H and N-H bonds, and that at 2895 cm^−1^ to the C-H bond. Additionally, absorption bands associated with the stretching vibrations of C-C (1441 cm^−1^) and C=N (1593 cm^−1^) bonds were obtained. NCDs’ surface was effectively modified with nitrogen and oxygen elements. Numerous C-O bonds are anticipated to improve the hydrophilicity of the NCDs, which may enhance their efficacy in detecting analytes in water-based solutions.

**Figure 2:**
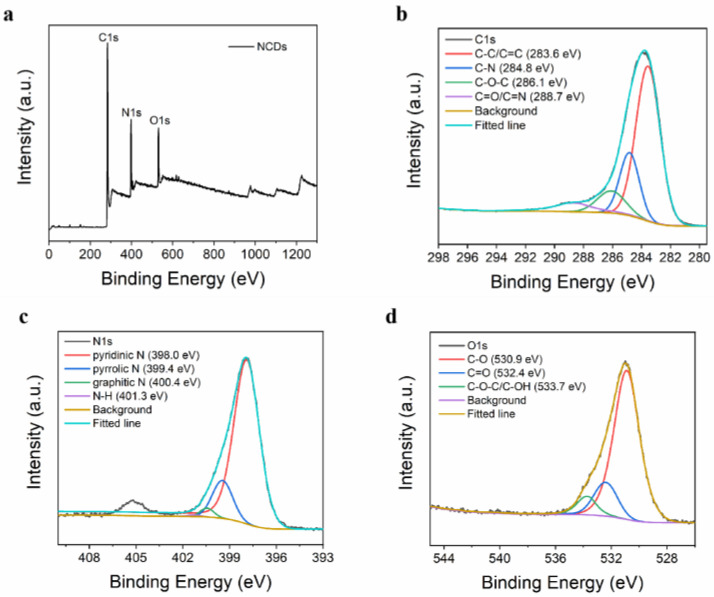
The full-scan XPS spectrum of NCDs (**a**), the C_1s_ XPS spectrum of NCDs with peak deconvolution (**b**), the N_1s_ XPS spectrum of NCDs with peak deconvolution (**c**), and the O_1s_ XPS spectrum of NCDs with peak deconvolution (**d**).

The chemical states of the constituent elements of NCDs were analyzed using XPS. The XPS spectra across the full range, as depicted in Figure 2a, revealed that the NCDs primarily consist of three elements: C (284.8 eV), N (398 eV), and O (530.5 eV). In Figure 2b, the bonds of C-C/C=C (283.6 eV), C-N (284.8 eV), C-O-C (286.1 eV), and C=O/C=N (288.7 eV) are represented by the peaks in the detailed C_1s_ spectrum. In Figure 2c, the N_1s_ spectrum was analyzed to show four different peaks at diverse binding energies: pyridinic N (398.0 eV), pyrrolic N (399.4 eV), N-H (401.3 eV), and pyrrolic N (400.4 eV) bonds. The redshift of PL emission is driven by the observed rise in graphite nitrogen content and the decrease in pyridine nitrogen content. The O1s spectrum in Figure 2d depicts the three oxygen types of NCDs: C-O (530.9 eV), C=O (532.4 eV), and C-O-C/C-OH (533.7 eV).

The characterization of the carbon materials of Lima’s team is relatively detailed [23], and the above characterization results of the synthesized carbon dots are compared with the earlier literature of the team, and the surface chemical structure of the synthesized carbon dots and the successful doping of nitrogen elements affect the luminescence of the carbon dots.

### 3.3. Optical Properties of NCDs

**Figure 3:**
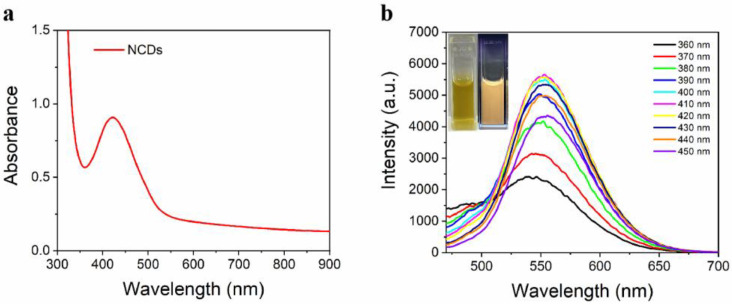
The UV–vis absorption spectra of NCDs (**a**) and the PL spectra of the NCDs at different excitation wavelengths (**b**).

The ultraviolet–visible absorption spectrum shown in Figure 3a reveals that the NCDs display a strong absorption peak at 420 nm. Carbon dots mainly absorb light in the UV range, with their absorption characteristics also being evident in the range of the visible spectrum. Electronic transitions like n-π*, p-π*, and π-π* inside aromatic ring structures with C=C and C=N/C=O bonds are associated with the absorption peaks of NCDs. Figure 3b shows the fluorescence emission spectra (using 5 μg·mL^−1^ NCDs) measured from 360 nm to 450 nm. The variation in peaks highlights the different excitation wavelengths. At 410 nm, the highest PL was recorded, peaking at 554 nm, demonstrating that NCDs display an emission behavior that is not influenced by the excitation wavelength.

Under natural light, the NCD solution exhibits a brown color, while under UV light, it displays a bright orange-yellow color. The optical stability of the prepared NCDs at room temperature and under UV lamp irradiation was further evaluated to assess their potential in practical applications. As illustrated in Figure S2a, NCDs have good fluorescence emission stability after 30 days of storage at room temperature. Furthermore, as illustrated in Figure S2b, the emission intensity of NCDs remained almost unaffected after 90 min of exposure to ultraviolet light irradiation, suggesting that the NCDs have good resistance to photobleaching. In Table S1, the QY of NCDs achieved a value of 28.41%; previous studies have established that the QY of water-soluble carbon dots generally falls within the 10–40% range. Impressively, the CDs prepared by our approach exhibit enhanced fluorescence emission characteristics relative to previously reported counterparts with similar QY values, making them highly conducive for potential analytical applications.

### 3.4. Luminescence Mechanism of NCDs

Modulating the doping process of carbon dots, especially nitrogen doping, serves as an efficient approach to improve quantum yield and enhance optical performance [24,25,26]. Herein, the precursors dihyne and 2-methylimidazole were utilized as the NCD sources.

**Figure 4:**
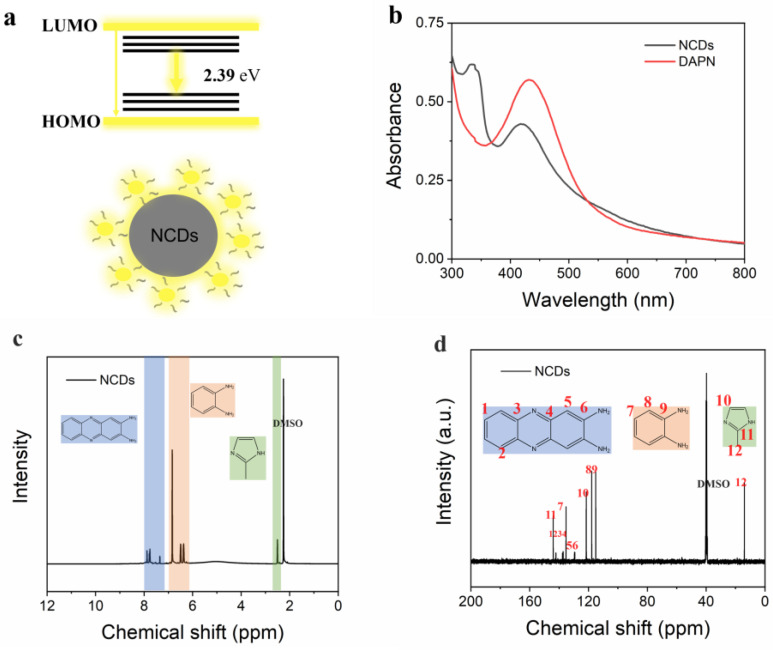
The energy gap of NCDs (**a**), the UV–vis absorption spectra of NCDs and 2,3-DAPN (**b**), the ^13^C NMR spectrum of NCDs (**c**), and the ^1^H NMR spectrum of NCDs (**d**).

The nitrogen in dihyne and 2-methyliopidazole that are used to synthesize the carbon dots in this study is graphite nitrogen and pyridine nitrogen. Alternatively, the optical band gap of carbon dots can be assessed by examining the absorption edge wavelength. As the graphitic nitrogen content rises, the separation between the HOMO and LUMO levels tends to decrease, thereby altering the optical behavior of the CDs. Many surface functional groups strongly moderate the highest occupied molecular orbital (HOMO) and lowest unoccupied molecular orbital (LUMO) levels of CDs. The HOMO/LUMO energy gap changes with size, thereby inducing significant changes in the absorption/emission spectra. The addition of electron-donating functional groups increases the HOMO and LUMO energy levels of the CDs, and the decrease in the HOMO energy is less than in the LUMO energy, leading to a smaller energy gap [23]. It is mathematically expressed as $E_{gopt}=1240/\lambda_{edge}$, where *λ_edge_* denotes the wavelength at the absorption edge’s maximum. As shown in Figure 4a, the calculation value is 2.39 eV, which can effectively tune the emission wavelength.

In order to further identify the fluorescent groups that triggered FL, the types of surface-oriented groups on the CDs were analyzed using ^13^C NMR and ^1^H NMR (Figure 4c,d). Chemistry displacement is consistent with the literature reports [24]. The hydrogen atoms on the phenylamine give rise to peaks at 6.84 ppm, 6.51 ppm, and 6.36 ppm in the ^1^H NMR spectrum. The peak at 2.51 ppm corresponds to the hydrogen on 2-methylimidazole. The peaks at 7.88 ppm, 7.64 ppm, and 7.34 ppm, as proven in the published literature, demonstrate that the characteristic chemical shift of 2,3-diosamone (2,3-DAPN) is consistent with the previously reported data [25]. In the ^13^C NMR spectrum, the peaks at 135.47 ppm, 118.96 ppm, and 115.18 ppm correspond to the carbon on phenylene. The peaks with chemical shifts of 144.32 ppm, 121.52 ppm, and 14.23 ppm correspond to the carbons on 2-methylimidazole. The peaks with chemical shifts of 142.36 ppm, 137.94–136.98 ppm, and 129.01–129.54 ppm were also observed, which belong to 2,3-DAPN. Based on the above comprehensive results, the carbon dot surface appears for 2,3-DAPN. Previous studies demonstrated that 2,3-diaminophenazine (2,3-DAPN) is generated through the oxidative transformation of phthalamine, which is subsequently adsorbed onto the surface of CDs.

In order to further explore whether 2,3-DAPN is an FL-transmitting group affecting the FL of NCDs, ultraviolet absorption spectroscopy was carried out. In Figure 4b, the n-π* electron transition of the aromatic ring structure is responsible for the distinctive absorption peak of 2,3-DAPN at around 429 nm. On the other hand, NCDs were found during the n-π* electron transition at 420 nm. 2,3-DAPN exhibits similar characteristic absorption behavior to NCDs, and this observation implies that the 2,3-diaminophenazine fluorophore likely serves as the primary contributor to the fluorescence emission through electron transition processes in NCDs upon visible light excitation.

Based on the reported literature, core states exhibit longer-lived components (>10 ns), while fluorophores show dominant short lifetimes (<5 ns). Moreover, carbon core emission demonstrates blinking-free behavior, and fluorophores exhibit characteristic on–off blinking. Core emission remains stable under mild oxidation, and fluorophore emission shows reversible changes with pH/modifiers [26,27]. The fluorescence lifetime of the NCDs (1.759 ns) aligns closely with the characteristic short lifetimes (<5 ns) of molecular fluorophores, contrasting with the longer lifetimes (>10 ns) expected from core state emission. The observed reversibility of fluorescence quenching and recovery and the pH-dependent changes at pH 2 and 7 (Figure S3) are characteristic behaviors of molecular fluorophores, as opposed to the stable emission and blinking-free behavior anticipated from core states. The primary fluorescence origin is confirmed from adsorbed 2,3-DAPN molecules. Based on the aforementioned investigations, it can be reasonably concluded that the fluorescence emission in NCDs is predominantly governed by molecular fluorophores like 2,3-DAPN, rather than intrinsic carbon core states.

### 3.5. Detection of NCDs Toward H_2_O_2_

**Figure 5:**
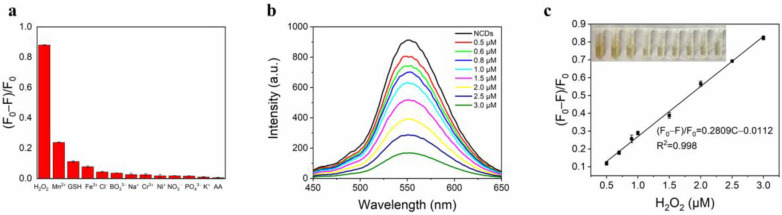
The impact of interfering ions (500 μM) on NCD fluorescence intensity (**a**), the fluorescence emission spectra of NCDs (5 μg·mL^−1^)/H_2_O_2_ (0.5 to 3 μM) (**b**), and the H_2_O_2_ concentration and NCDs fluorescence quenching have a linear relationship in the range of 0.5–3 μM (**c**).

Owing to their superior photoluminescent characteristics, NCDs have become promising candidates for fluorescent probe applications, wherein exceptional selectivity represents a fundamental requirement. In this experiment, the observed quenching of NCDs’ fluorescence emission upon H_2_O_2_ exposure demonstrates their promising applicability. To evaluate the selective sensing capability of the synthesized NCDs toward H_2_O_2_, systematic investigations were conducted on the fluorescence response profiles of various potential interfering species, maintaining a consistent analyte concentration of 500 μM. The fluorescence intensities were denoted as F_0_ and F, respectively. As revealed in Figure 5a, only H_2_O_2_ can significantly reduce the fluorescence of NCDs. The results indicated that NCDs possess good chemical stability and selectivity, emphasizing their potential for detecting H_2_O_2_.

The detection sensitivity was quantitatively assessed by monitoring the fluorescence response of NCDs to the incremental additions of H_2_O_2_. Figure 5b clearly demonstrates a dose-dependent quenching effect, where the emission intensity of NCDs exhibits a gradual attenuation proportional to the H_2_O_2_ concentration. The fluorescence quenching response showed a well-defined linear dependence on H_2_O_2_ concentration in the 0.5–3 μM range, as illustrated in Figure 5c, and the correlation coefficient was extremely high (R^2^ = 0.998). The relationship was quantified using the fluorescence intensities of NCDs and NCDs/H_2_O_2_ represented by F_0_ and F in the linear regression equation (F_0_ − F)/F_0_ = 0.2809C − 0.0112. The 3δ criterion (LOD = 3δ/K) yielded a detection limit of 0.28 μM, where δ represents standard deviation and K represents slope. In comparison with other previously reported literature (Table 1), the LOD achieved with this method is relatively low.

### 3.6. Detection of AA

**Figure 6:**
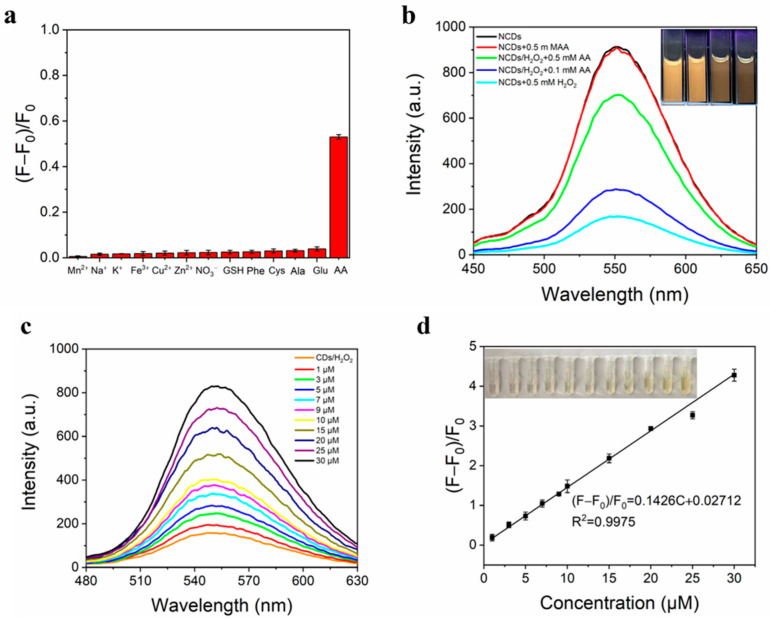
The investigation on the performance of CDs/H_2_O_2_ mixed system (**a**), the fluorescence “on–off–on” behavior of NCDs upon the sequential addition of H_2_O_2_ and AA (**b**), the fluorescence emission spectra of NCDs/H_2_O_2_ system with varying concentrations of AA (1–30 µM) (**c**), and the linear relationship between (F − F_0_)/F_0_ and AA concentration in the range of 1–30 µM (**d**).

Considering the fluorescence quenching of CDs by H_2_O_2_, a series of experiments were conducted to evaluate the H_2_O_2_ detection performance. Different ions and substances were added to the CDs/H_2_O_2_ system to investigate fluorescence recovery. Each group was tested in triplicate with the same ion concentration. From Figure 6a,b, results showed that AA could restore the fluorescence of CDs. Further experiments were performed to analyze AA using fluorescence detection. Figure 6c illustrates the dose-responsive fluorescence enhancement of the NCDs/H_2_O_2_/AA system following the introduction of AA solutions with increasing concentrations. As depicted in Figure 6d, the normalized fluorescence intensity (F − F_0_)/F_0_) and ascorbic acid concentrations ranging from 1 to 30 μM were shown to be linearly related. The correlation coefficient R^2^ was found to be 0.997. The calibration curve was derived using the linear regression equation (F − F_0_)/F_0_ = 0.1428C − 0.0112, resulting in an LOD of 0.605 μM. This LOD was calculated using the same method applied for the quantification of H_2_O_2_.

To demonstrate the sensitivity of AA detection, Table 2 compares the LOD of AA detection using this method with those previously reported in the literature, revealing that the detection limit of this method is relatively low. Kawan F. Kayani’s team used a one-step hydrothermal synthesis method with 6,9-diamino-2-ethoxyacridine lactate as the precursor. The introduction of Fe^3+^ and AA resulted in both fluorescence quenching and the enhancement of the synthesized N-CDs. The fluorescence of the N-CDs was recovered upon the addition of AA to the N-CDs-Fe^3+^ system. Using the “off–on” fluorescent N-CD probe, a linear range of 40–90 µM was achieved with an LOD of 0.69 µM [33]. Gu’s team prepared nitrogen-doped carbon quantum dots (N-CQDs) with wolfberry. These N-CQDs were developed as a highly sensitive fluorescent “on–off–on” switch sensor for the sensing of Fe^3+^ and AA. The addition of AA is supposed to repair the surface defects, resulting in the fluorescence recovery. Based on this effect, the strategy of “on–off–on” detection of AA was established with a limit of detection at 1.8 μM [34]. Yadav’s team prepared carbon quantum dots using the one-pot hydrothermal treatment of leaf extracts of neem (Azadirachta indica). The as-synthesized neem carbon quantum dots (N-CQDs) exhibited up to 27.2% of highly fluorescent QYs. The linear range of AA detection was between 5 and 40 μM with an LOD of up to 1.773 μM [18]. Wei’s team prepared carbon dots using microwave irradiation and electropolymerization on a glassy carbon electrode (GCE) to establish an electrochemical sensor for the selective detection of AA. The wide linear responses were obtained in the ranges of 0.01–3 mM and 4–12 mM with a detection limit of 10 μM to AA [35]. Li’s team fabricated cobalt-doped carbon quantum dots (Co-CQDs) using a one-pot hydrothermal method with cobalt tetraphenylporphyrin and 1,2-ethanediamine as precursors. CDs can act as a fluorescent probe for the detection of Fe^3+^ and AA with high selectivity and sensitivity through an “on−off−on” mode. The quenched emission of carbon quantum dots can be recovered with the addition of AA to the Co-CQDs/Fe^3+^ system. The change in fluorescence with the concentration of AA (0.6−1.6 mM) was linear, with an LOD of 18 μM [36]. Overall, these results underscore the method’s high sensitivity and potential for accurate AA detection in practical applications. A fluorescence-based “off–on” probe utilizing NCDs was developed for AA sensing and detection.

To further investigate the applicability of this technology, four types of commercially available vitamin C-containing beverages were detected to validate the concentration of AA in the genuine samples, followed by spiked recovery experiments to confirm the method’s accuracy. Table 3 reveals that the spiking recovery rate ranged from 95.9% to 100.2%, indicating a satisfactory spike recovery effect. This result demonstrates that NCDs can be utilized to detect AA in real samples.

### 3.7. Quenching Mechanism of Fluorescence “Off” and “On”

**Figure 7:**
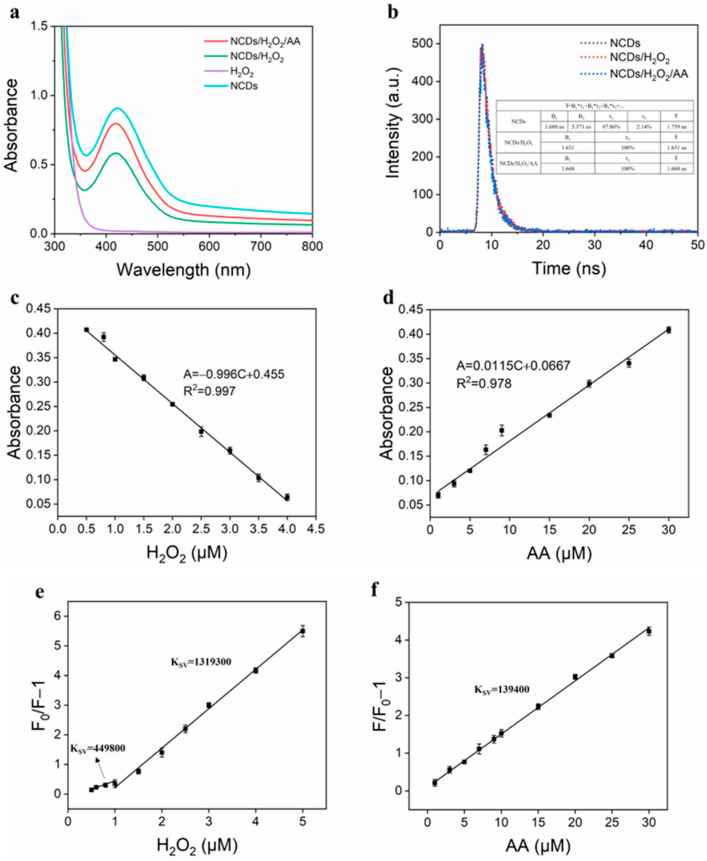
The UV–vis absorption spectra of NCDs, H_2_O_2_, NCDs/H_2_O_2_, and NCDs/H_2_O_2_/AA (**a**), (**b**) the fluorescence emission decay curves of NCDs, NCDs/H_2_O_2_, and NCDs/H_2_O_2_/AA system (lex/lem = 410/554 nm) (**b**), the absorbance of NCDs/H_2_O_2_ (0.5–5 μM) (**c**), the absorbance of NCDs/H_2_O_2/_AA (1–30 μM) (**d**), the Stern–Volmer curves for the suppression of NCD fluorescence at varying H_2_O_2_ (0.5–1 μM 1–5 μM) concentrations (0.5–5 μM) (**e**), and the Stern–Volmer plots for the fluorescence recovery of the NCDs/H_2_O_2_/AA system (1–30 μM) (**f**).

The fluorescence quenching phenomenon involves intricate processes that generally encompass both static and dynamic quenching pathways [37], which generate a ground-state charge-transfer complex, and static quenching is caused by the interaction of the fluorophore with the quencher. In contrast, the dynamic quenching process involves fluorescence deactivation via diffusional encounters between the quenching species and the excited luminophore [38]. To better study the fluorescence quenching mechanism in this system, a comprehensive set of experimental studies was performed. The discrimination between these two quenching processes was primarily accomplished by examining the ultraviolet–visible absorption spectral characteristics of NCDs under different H_2_O_2_ concentrations [39]. As mentioned, the NCDs exhibited a reversible “off–on” fluorescence response upon the sequential addition of H_2_O_2_ and ascorbic acid (AA) to their aqueous dispersion, respectively. Figure 7a demonstrates the UV–vis absorption spectral analysis, indicating that the characteristic absorption band intensity of NCDs exhibits a significant reduction upon H_2_O_2_ introduction. However, when AA is introduced into the NCDs/H_2_O_2_ mixed system, the absorption peak recovers. Under photoexcitation, electrons on the NCDs undergo transitions, and the injection of H_2_O_2_ causes growth defects on the surface of NCDs, which are then successfully passivated by the insertion of AA. These photoexcited electrons tend to return to the ground state, facilitating the recovery of excited-electron transitions in NCDs. Additionally, the optical absorption spectra were systematically recorded to quantify the absorbance characteristics of the NCDs/H_2_O_2_ systems with varying H_2_O_2_ concentrations, followed by the spectroscopic evaluation of AA concentration-dependent absorbance modulation in the NCDs/H_2_O_2_/AA ternary systems. As the concentration of H_2_O_2_ increases, a gradual decrease in absorbance is observed, adhering to the linear equation A = −0.996C + 0.455. Simultaneously, the introduction of varying concentrations of ascorbic acid (AA) into the NCDs/H_2_O_2_ composite system exhibits a pronounced linear correlation, represented by the equation A = 0.0115C + 0.0667 (Figure 7c,d). The higher slope of this equation compared to the previous one further indicates that the system is primarily controlled by static quenching processes.

Crucially, unlike dynamic quenching, static quenching does not affect the fluorescence lifetime (τ₀). This is because the fluorophore is effectively removed from the excited state population through complex formation rather than collisional deactivation. Consequently, the ratio of the fluorescence lifetime in the absence of a quencher (τ₀) to the fluorescence lifetime in the presence of a quencher (τ) is unity (τ₀/τ = 1).

Time-resolved fluorescence spectroscopy employing the TCSPC methodology was utilized to probe the excitation dynamics and energy transfer mechanisms in carbon-based quantum dots [40] and to determine the fluorescent decay curves of NCDs, NCDs/H_2_O_2_, and NCDs/H_2_O_2_/AA. In Figure 7b, the fluorescence lifetime of NCDs is 1.759 ns (Table S2), which remains essentially unchanged following interaction with H_2_O_2_. After interacting with AA, the fluorescence lifetime of the NCDs/H_2_O_2_ combination stays constant, suggesting static quenching.

Static quenching is characterized by distinct features. A key indicator is a linear Stern–Volmer plot, where the ratio of initial fluorescence intensity to fluorescence intensity in the presence of a quencher is plotted against the quencher concentration. This plot yields a straight line with a slope equal to the static quenching constant.

In this experiment, the fluorescence intensity of NCDs is expected to vary with the concentration of H_2_O_2_ in accordance with the Stern–Volmer equation: F_0_/F − 1 = K_sv_ [Q] = k_q_τ_0_ [Q]. In this equation, [Q] represents the molar concentration of hydrogen peroxide, K_sv_ represents the Stern–Volmer quenching constant, τ_0_ represents the intrinsic fluorescence lifetime of NCDs without quenching, k_q_ represents the bimolecular quenching rate constant, and F_0_ and F represent the steady-state fluorescence intensities of NCDs with and without a quencher, respectively.At the same time, we subsequently investigated the Stern–Volmer quenching kinetics between the NCDs/H_2_O_2_ system and the varying concentrations of ascorbic acid, in which F_0_, F, [Q], K_sv_, k_q_, and τ_0_ corresponded to the NCDs/H_2_O_2_/AA mixture, respectively. From the data presented in Figure 7e, it is evident that the relationship between NCDs’ fluorescence emission and H_2_O_2_ concentration adheres to the Stern–Volmer equation within both the ranges of 0.5–1.0 μM and 1.0–5.0 μM. The K_sv_ is 44,500 and 18,640 mol^−1^ L, respectively, for these two concentration ranges. Additionally, their k_q_ is 2.6 × 10^14^ and 8.4 × 10^14^ mol L^−1^ s^−1^, respectively. The dependence of the NCDs/H_2_O_2_ system’s fluorescence emission on the AA concentration is linear from 1 to 30 μM, which conforms with the Stern–Volmer equation, as shown in Figure 7f, with a K_sv_ of 139,400 mol^−1^ L and a k_q_ of 1.01 × 10^14^ mol L^−1^ s^−1^. It is well established that the maximum dynamic quenching rate constant of quenchers typically does not exceed 10^10^ mol L^−1^ s^−1^ [41]. Compared to this maximal quenching rate constant, the quenching rate constant in this experiment is substantially bigger, and the quenching behavior suggests a static quenching mechanism, as opposed to dynamic quenching.

The fluorescence quenching by H_2_O_2_ proceeds through a static quenching-dominated process, as evidenced by the following: a linear Stern–Volmer relationship at low H_2_O_2_ concentrations, unaffected fluorescence lifetimes, ruling out dynamic quenching, and UV–vis absorption confirming ground-state complexation. H_2_O_2_ selectively targets electron-rich surface fluorophores and oxidizes edge carboxyl groups on the carbon core. AA reduces oxidized fluorophores via a two-electron transfer, and the restoration of π-conjugation in fluorophores revives an intramolecular charge transfer. AA scavenges H_2_O_2_-generated -OH radicals, preventing further carbon core degradation, and carboxyl group reduction reverses surface oxidation.

The experimental results suggest a static quenching mechanism wherein H_2_O_2_ interacts with the NCDs to form a complex that quenches fluorescence. The subsequent introduction of AA triggers a reaction with H_2_O_2_ (AA + H_2_O_2_ → DHAA + 2H_2_O) [42,43], consuming the quencher and leading to the desorption of H_2_O_2_ from the NCD surface, thus restoring fluorescence. As an antioxidant, AA reduces H_2_O_2_ via electron donation and is oxidized to DHAA. The presence of carboxyl and hydroxyl groups on the NCD surface may facilitate its interactions with AA or its oxidation products. Thus, the observed fluorescence recovery can be attributed to a competitive interfacial redox process as follows: initially, H_2_O_2_ quenches NCD fluorescence via an electron transfer from NCDs to H_2_O_2_. Subsequently, AA preferentially reduces H_2_O_2_, and is oxidized to DHAA. Therefore, the introduction of AA into the NCD system could inhibit electron transfers from NCDs to H_2_O_2_, resulting in the fluorescence recovery of NCDs.

The electronic mechanism between NCDs and AA can be described as follows: First, an electron transfer occurs from NCDs to H_2_O_2_. This can happen via two pathways: an electron transfer from the NCDs’ conduction band to the LUMO of H_2_O_2_, or an electron transfer from the surface states on NCDs to H_2_O_2_. This electron transfer process creates an electron depletion layer on the NCD surface, effectively reducing the number of available electrons for radiative recombination and leading to fluorescence quenching. Second, AA facilitates fluorescence recovery by effectively injecting holes into the H_2_O_2_-occupied surface states on the NCDs. AA donates electrons, undergoing oxidation to form a radical intermediate (AA•): AA → AA• + e⁻. This electron effectively fills the surface traps created by H_2_O_2_ quenching, neutralizing the positive charge buildup and partially restoring the original electronic structure of the NCD surface. The AA• further oxidizes to DHAA, releasing protons and electrons: AA• → DHAA + 2H⁺ + e. This second electron donation step further regenerates the NCD surface by removing the trapped positive charges and restoring the original electron density.

In summary, the synthesized NCDs exhibit potential as bifunctional probes, capable of identifying both H_2_O_2_ and AA.

## 4. Conclusions

In this study, highly fluorescent NCDs were successfully fabricated through the hydrothermal approach, employing *o*-phenylenediamine and 2-methylimidazole as dual precursors for carbon and nitrogen doping. For the measurement of H_2_O_2_ and AA, a fluorescence “off–on” system based on the NCDs was effectively created. The sensing mechanism involves the selective oxidation of the NCD surface by H_2_O_2_, leading to fluorescence quenching; this phenomenon is subsequently reversed upon ascorbic acid (AA) introduction, attributed to the surface defect passivation through reduction processes. Compared to traditional nanosensors, the NCD-based “off–on” fluorescence probes offer several advantages, including ease of synthesis, excellent optical properties, and high selectivity and specificity. The successful detection of AA in commercial beverages using the synthesized NCDs highlights their promising potential for real-world analytical applications. However, the current experimental validation, primarily focusing on these beverage samples, presents some limitations regarding the complexity of real-world matrices. Future studies should expand the application of these NCDs to more complex biological (e.g., serum, urine, etc.) or environmental (e.g., river water, soil extracts, etc.) matrices. This application expansion would provide a more comprehensive assessment of the NCDs’ selectivity, sensitivity, and overall suitability for a broader range of real-world analytical challenges. Beyond matrix expansion, future investigations could also focus on enhancing the sensitivity and robustness of the NCDs, broadening their applicability to the detection of other relevant analytes, and developing portable sensing devices for convenient on-site analysis.

## Figures and Tables

**Table 1:** The proposed H_2_O_2_ detection method’s analytical performance comparisons with other published methods.

Material	Linear Range (µM)	LOD (µM)	References
CeO_2_-CDs	1.67–2.01	0.35	[28]
Cs-oPD-CDs	1–200	0.51	[29]
Cu, Cl-CDs	1–600	0.35	[30]
N, Fe-CDs	1–200	0.40	[31]
N, S-CDs	10–140	1.9	[32]
NCDs	0.5–3	0.28	This work

**Table 2:** The analytical performance comparison of the proposed method for AA detection with other CD-based methods reported in the literature.

Probe	Linear Range	LOD (µM)	References
6,9-diamino-2-ethoxyacridine-CDs	40–90 µM	0.69 µM	[33]
Wolfberry-CDs	10–100 µM	1.80 µM	[34]
Leaf extracts of neem-CDs	5–40 µM	1.773 µM	[18]
DA-UA-CDs	0.01–3 mM	10 µM	[35]
Co-CDs	0.6–1.6 mM	18 µM	[36]
NCDs	1–30 µM	0.605 µM	This work

**Table 3:** The evaluation of AA content in drink samples employing the suggested approach.

Beverage Samples	Dilution	Added AA Amount (µM)	Fluorometric (µM)	Recovery (%)	RSD (n = 3, %)
Alienergy Vitamin C	200	0	0.3		
		20	19.48	95.9%	0.56%
		25	25.10	99.2%	1.1%
Nongfu Spring C100	200	0	0.62		
		20	20.39	98.9%	0.87%
		25	25.50	99.6%	0.45%
Lemon Soda	150	0	0.52		
		20	20.56	100.2%	0.85%
		25	25.15	98.5%	0.35%
Minute Maid Orange	50	0	0.37		
		20	20.18	99.1%	1.3%
		25	25.23	99.5%	0.67%

## Data Availability

Data are contained within the article.

## Supplementary Materials

The following supporting information can be downloaded at: https://www.mdpi.com/article/10.3390/nano15130976/s1, Figure S1: The fluorescence emission spectra of NCDs with different (a) reaction times and (b) reaction temperatures. Figure S2: (a) The continuous fluorescence intensity measurements of NCD aqueous solutions in 30 days. (b) The fluorescence stability of NCDs in an aqueous solution with different UV light irradiation times. Figure S3: The reversible response of NCDs to pH. Table S1: The parameters of fluorescence quantum yield of NCDs. Table S2: The computational process of $\bar{\tau }$ of the NCDs.
