# Supplementary material for: Nitrogen-Doped Carbon Dots as a Fluorescent “Off–On” Probe for Selective Ascorbic Acid Detection via H2O2-Mediated Quenching
Source: Nanomaterials (Basel). 2025 Jun 23;15(13):976. doi: 10.3390/nano15130976 (PMC12250831; doi:10.3390/nano15130976)
Supplement: Supplementary file 1 [file nanomaterials-15-00976-s001.zip › nanomaterials-3668526-supplementary.pdf]

Supplementary Materials

Nitrogen-Doped Carbon Dots as a Fluorescent “Off–On” Probe for Selective Ascorbic Acid Detection via H_2O_2-Mediated Quenching

Jingjing Jia, Xue Liu and Wenjing Wang *

College of Chemistry and Chemical Engineering, Shandong Sino-Japanese Center for Collaborative Research of Carbon Nanomaterials, Instrumental Analysis Center of Qingdao University, Qingdao University, Qingdao 266071, China; 2022020328@qdu.edu.cn (J.J.); 2023020334@qdu.edu.cn (X.L.)

* Correspondence: wangwenjing@qdu.edu.cn; Tel.: 86-532-85953981

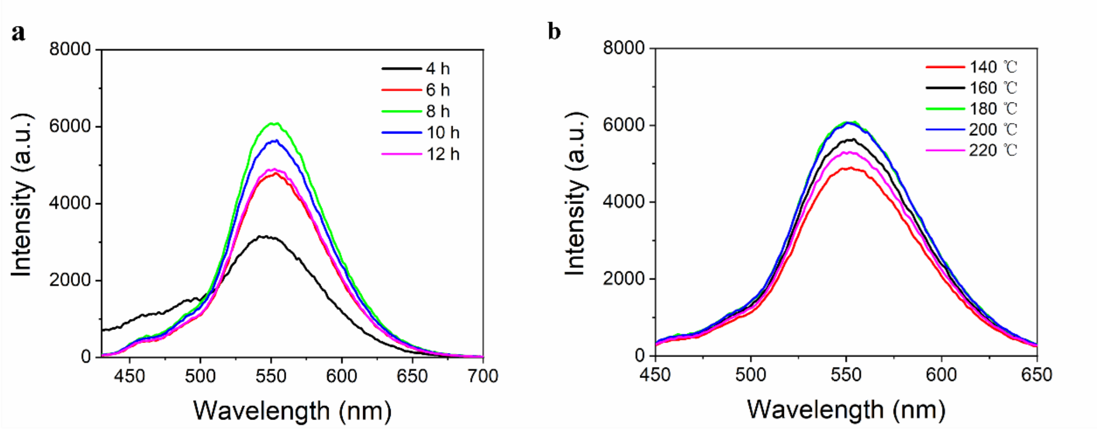

Figure S1. The fluorescence emission spectra of NCDs with different (a) reaction times, (b) reaction temperatures.

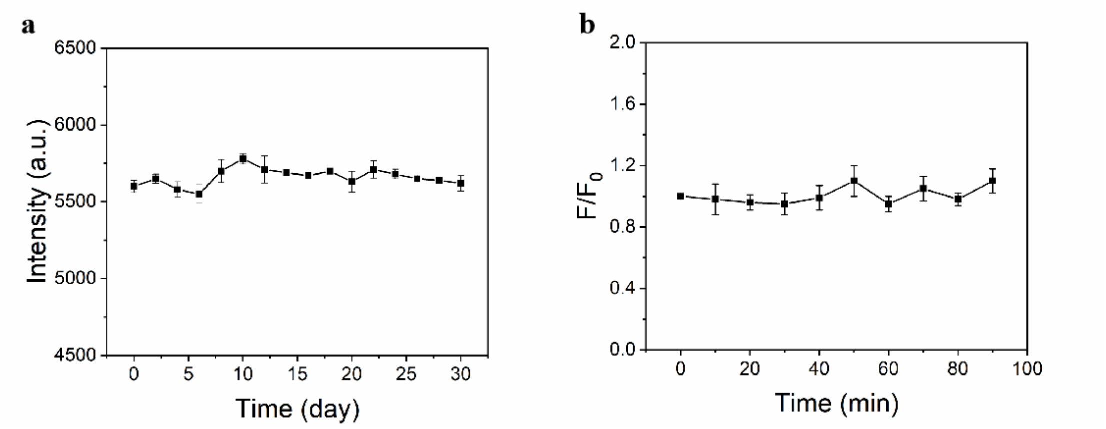

Figure S2. (a) The continuous fluorescence intensity measurements of NCDs aqueous solutions in 30 days. (b) Fluorescence stability of NCDs in aqueous solution with different irradiation time by UV light.

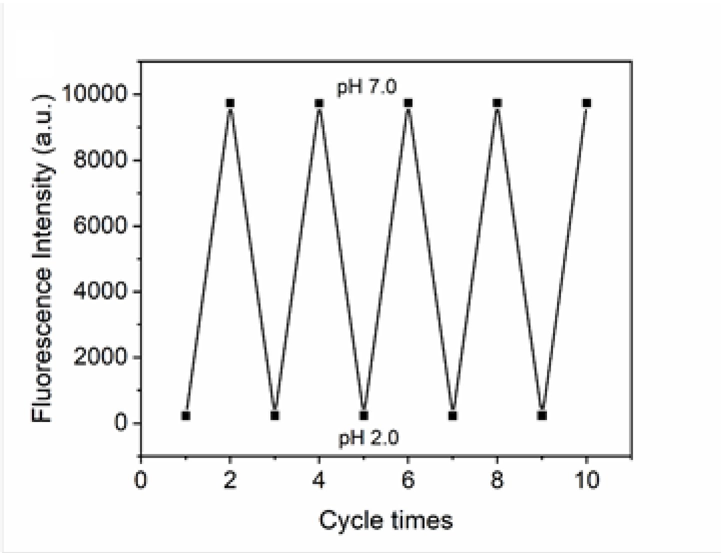

Figure S3. Reversible response of NCDs to pH.

Table S1. Parameters of fluorescence quantum yield of NCDs.

	**Fluorescence integral area**	**Absorbance**	**QY**
Quinine Sulfate	57166.190	0.045	56%
NCDs	239170.112	0.0059	28%

Table S2. The computational process of $\bar{\tau}$ of the NCDs.

**$\bar{\tau}$=B_1*τ_1+B_2*τ_2+B_3*τ_3+...**					
**NCDs**	**B_1**	**B_2**	**τ_1**	**τ_1**	$\bar{\tau}$
	1.680 ns	5.371 ns	97.86%	2.14%	1.759 ns
$NCDs/H_2O_2$	B_1		τ_1		$\bar{\tau}$
	1.631		100%		1.631 ns
$NCDs/H_2O_2/AA$	B_1		τ_1		$\bar{\tau}$
	1.668		100%		1.668 ns

According to the below equation and related parameters in Table S2.

$$\boldsymbol{R(t) = B_1 e^{(-\tau/\tau_1)} + B_2 e^{(-\tau/\tau_2)} + B_3 e^{(-\tau/\tau_3)}}.$$

Table 2. f N-CDs, N-CDs/H_2O_2, and N-CDs/H_2O_2/AA are as follows:.

NCDs: $\boldsymbol{R(t)} = \boldsymbol{6666.2}407e^{(-\tau/1.627)} + 12.3896\, e^{(-\tau/9.345)}$, $\chi^2 = 1.2879$.

NCDs/H_2O_2: $\boldsymbol{R(t)} = \boldsymbol{441.2}453e^{(-\tau/4.605)} + 114.1003\, e^{(-\tau/2.139)}$, $\chi^2 = 1.2118$.

NCDs/H_2O_2/AA: $\boldsymbol{R(t)} = \boldsymbol{461.4}502\, e^{(-\tau/3.943)} + 114.6689\, e^{(-\tau/2.363)}$, $\chi^2 = 1.1436$.
